# Anticorrosive Effects of Some Thiophene Derivatives Against the Corrosion of Iron: A Computational Study

**DOI:** 10.3389/fchem.2018.00155

**Published:** 2018-05-07

**Authors:** Lei Guo, Zaki S. Safi, Savas Kaya, Wei Shi, Burak Tüzün, Nail Altunay, Cemal Kaya

**Affiliations:** ^1^School of Material and Chemical Engieering, Tongren University, Tongren, China; ^2^Chemistry Department, Faculty of Science, Al Azhar University-Gaza, Gaza City, Palestine; ^3^Department of Chemistry, Faculty of Science, Cumhuriyet University, Sivas, Turkey

**Keywords:** corrosion inhibition, molecular dynamics simulation, DFT, adsorption, principle component analysis

## Abstract

It is known that iron is one of the most widely used metals in industrial production. In this work, the inhibition performances of three thiophene derivatives on the corrosion of iron were investigated in the light of several theoretical approaches. In the section including DFT calculations, several global reactivity descriptors such as *E*_HOMO_, *E*_LUMO_, ionization energy (*I*), electron affinity (*A*), HOMO-LUMO energy gap (Δ*E*), chemical hardness (η), softness (σ), as well as local reactivity descriptors like Fukui indices, local softness, and local electrophilicity were considered and discussed. The adsorption behaviors of considered thiophene derivatives on Fe(110) surface were investigated using molecular dynamics simulation approach. To determine the most active corrosion inhibitor among studied thiophene derivatives, we used the principle component analysis (PCA) and agglomerative hierarchical cluster analysis (AHCA). Accordingly, all data obtained using various theoretical calculation techniques are consistent with experiments.

## Introduction

**Figure 1:**
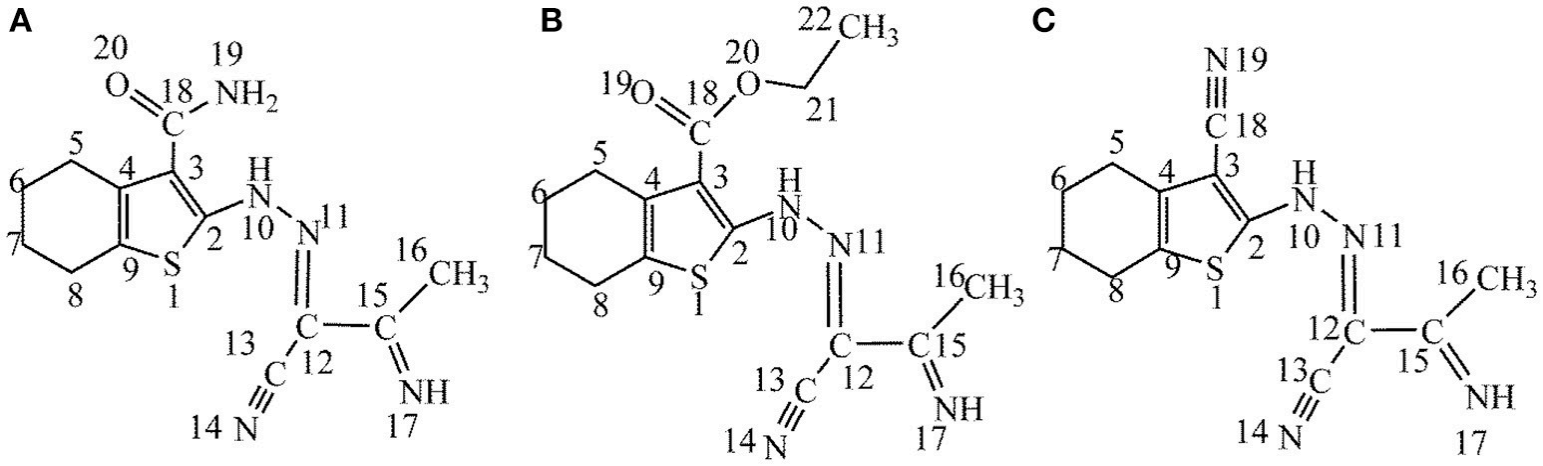
**(A–C)** Chemical molecular structures of studied thiophene derivatives (the number indicates atom-numbering).

One of the serious problems in industrial sector is the corrosion of metals or alloys, which causes great casualties and enormous property loss (Frankel et al., 2015; Li et al., 2015). The most environmentally-benign and cost-effective approach to prevent metals against corrosion in acid solutions is using of inhibitors (Raja et al., 2016). Organic molecules containing O, N, and/or S atoms are the most widely used and are considered to be effective corrosion inhibitors (Xhanari et al., 2017). It is generally assumed that they can be adsorbed at metal surface through some active groups like heteroatoms, triple bonds or aromatic rings (Kovačević and Kokalj, 2013; Ko and Sharma, 2017). Previous studies showed that most organic inhibitors decrease corrosion rate by adsorption on the substrate surface and the inhibition performance follows the sequence O < N < S < P (Loto et al., 2012). Bockris and Swinkels suggested that S and/or N atoms could easily adsorb on metal surface by replacing water molecules (Bockris and Swinkels, 1964). The interactions between the organic corrosion inhibitors and the metal substrates are generally divided into two types: physical adsorption and chemical adsorption. On the basis of the theory hard and soft acids and bases (HSAB) introduced by Pearson (Pearson and Songstad, 1967), molecules including sulfur atom in their molecular structures can be regarded as soft bases, which give easily electron to metal surfaces because they cannot resist to electron cloud polarization or deformation. Thiophene is a sulfur containing heterocyclic compound with the molecular formula C_4_H_4_S. The electron pairs on sulfur atom are delocalized in π-conjugated systems. Thiophene and its derivatives can be obtained from petroleum or coal and in general are well-known because of their therapeutic applications in medicinal chemistry. Many theoretical and experimental studies including the analyzing of inhibition efficiencies of thiophene and its derivatives are available in the literature (Benabdellah et al., 2011; Gece, 2013; Yadav et al., 2014). Especially, Fouda et al. (2014) synthesized three thiophene derivatives and investigated their anticorrosive effects against the corrosion of carbon steel using experimental methods such as weight loss, Tafel test, electrochemical frequency modulation, and electrochemistry impedance test. The molecular structures of mentioned molecules are given in Figure 1.

It is well-known that reactivity in chemistry is a back-bone because it is well associated with reaction mechanisms. Therefore it allows us to understand chemical reactions, behavior of substance and improving synthesis procedures to obtain new materials such as corrosion inhibitors or drugs. Quantum chemistry calculations have proven to be very effective in evaluating the corrosion inhibition efficiency (Khalil, 2003; Obot et al., 2015; Taylor, 2015; Lgaz et al., 2018). They have been widely employed to interpret the experimental phenomena. Especially in recent years, conceptual density functional theory (CDFT) has been developed and applied to analyze the molecular activity of inhibitors (Geerlings et al., 2003; Liu, 2009). Furthermore, molecular dynamics simulation has become another effective way to explore the structure and bonding characteristics at inhibitor/metal interface (Khaled, 2008; Oguzie et al., 2013; Wang et al., 2016).

In the present work, three earlier mentioned thiophene derivatives (see Figure 1), namely, 2-[(2Z)-2-(1-cyano-2-iminopropylidene)hydrazinyl]-4,5,6,7-tetrahydro-1-benzothiophene-3-carboxami de (A), ethyl-2-[(2Z)-2-(1-cyano-2-iminopropylidene)hydrazinyl]-4,5,6,7-tetrahydro-1-benzothiophe ne-3-carboxylate (B), as well as 2-[(2Z)-2-(1-cyano-2-iminopropylidene)hydrazinyl]-4,5,6,7-tetrahy dro-1-benzothiophene-3-carbonitrile(C), have been chosen to theoretically analyze their anticorrosive efficiencies. The objective of the work is to assess their anti-corrosive performances of studied molecules applying quantum chemical calculations, molecular dynamics simulation, principle component analysis (PCA) as well as agglomerative hierarchical cluster analysis (AHCA).

## Computational methods and definitions

### Gaussian calculations

Gaussian 09 package program was used to the study of the isolated compounds using density functional theory (DFT), with the B3LYP functional (Wiberg, 2004) under the SDD, 6-31G and 6-31++G basis sets. For comparison, the calculations based on Hartree–Fock (HF) theory were also performed. Gauss View 5.0.8 program was employed to prepare the correlative calculation parameters for the compounds under probe.

As we all know, electrochemical corrosion generally happens in liquid environment. One of the most popular approaches to research solvent effect is to consider hydrogen bonded clusters of solvent molecules surrounding the solute molecules. Thus self-consistent reaction field (SCRF) theory, with Tomas's polarized continuum model (PCM) (Andzelm et al., 1995) was used to describe the solvent effect of water. This method describes the solvent as a structureless continuum with uniform dielectric permittivity, in which a molecular-shaped empty cavity is dug to host the solute (Scalmani et al., 2006; Aouniti et al., 2013). The reliability of PCM method to explore the solvent effect in the field of corrosion inhibitors has been validated by many researchers (Wazzan et al., 2016; Guo et al., 2017; Yang et al., 2017).

### Global reactivity descriptors

Chemical reactivity can be simply defined as the tendency of a chemical matter to undergo chemical reaction with another chemical matter. It is well-known that the understanding of the nature of chemical interactions and the prediction of chemical reactivity of atoms, ions or molecules are some of the challenging issues in chemistry. In the CDFT, quantum chemical descriptors like electronegativity (χ), chemical hardness (η), and chemical potential (μ) are usually considered. μ and η are defined as the first derivative of the electronic energy and chemical potential with respect to the electron number (*N*) at constant external potential, *v*_(r)_, respectively (Liu, 2009; Frau and Glossman-Mitnik, 2017).

(1) $$\mu ={\left(\frac{\partial E}{\partial N}\right)}_{\nu (r)}$$

(2) $$\eta =\frac{1}{2}{\left(\frac{\partial^{2}E}{\partial N^{2}}\right)}_{\nu (r)}=\frac{1}{2}{\left(\frac{\partial \mu }{\partial N}\right)}_{\nu (r)}$$

Within the framework of finite differences approximation, the following expressions based on first vertical ionization energy and electron affinity values of chemical species are given (Madkour and Elshamy, 2016).

(3) $$\chi =-\mu =\left(\frac{I+A}{2}\right)$$

(4) $$\eta =\frac{I-A}{2}$$

Softness that is known as a measure of the polarizability is mathematically defined as the multiplicative inverse of chemical hardness:

(5) σ=1/η

Ionization energies and electron affinities of molecules can be predicted *via* Koopman's Theorem (Bellafont et al., 2015), i.e., ionization energy and electron affinity values of a chemical species correspond to negative values of its HOMO and LUMO orbital energies, respectively. So we can write the following equations for the calculation of quantum chemical parameters like hardness, electronegativity and chemical potential.

(6) $$\chi =-\mu =\left(\frac{-E_{\text{HOMO}}-E_{\text{LUMO}}}{2}\right)$$

(7) $$\eta =\left(\frac{E_{\text{LUMO}}-E_{\text{HOMO}}}{2}\right)$$

Besides, global electrophilicity index (ω) introduced by Parr (Parr et al., 1999), nucleophilicity (ε), which is physically the inverse of the electrophilicity, the fraction of electrons (Δ*N*) transferred from (to) the inhibitor molecule to (from) the metal surface, the energy of back donation Δ*E*_b-d_, electronic charge accepting capability and the initial molecule-metal interaction energy Δψ, were calculated in terms of global hardness (η) and electronegativity (χ) as given in Equations (8–12).

(8) $$\omega =\mu^{2}/2\eta \;=\chi^{2}/2\eta$$

(9) ε=1/ω

(10) $$\Delta N=\frac{\chi_{Fe}-\chi_{inh}}{2(\eta_{\text{Fe}}+\eta_{\text{inh}})}$$

(11) $$\Delta E_{back-donation}=-\frac{\eta }{4}=\frac{1}{8}\left(E_{\text{HOMO}}-E_{\text{LUMO}}\right)$$

(12) $$\Delta \psi =-\frac{{\left(\chi_{\text{Fe}}-\chi_{\text{inh}}\right)}^{2}}{4\left(\eta_{\text{Fe}}+\eta_{\text{inh}}\right)}$$

Herein, χ_Fe_, χ_inh_, η_Fe_, and η_inh_ represent the absolute electronegativity and hardness of iron and inhibitor molecule, correspondingly. In order to obtain the Δ*N* values, we adopted a theoretical value of χ_Fe_ = 7.0 eV and η_Fe_ = 0 by assuming that for a metallic bulk *I* = *A* since they are softer than the neutral metallic atoms (Zarrouk et al., 2014).

### Fukui functions

Evaluation of the Fukui functions has been employed to explore the local reactivity of the molecules. Yang and Mortier (1986) defined the Fukui function as the first derivative of the electronic density ρ(*r*) of a system with respect to the number of electrons (*N*) at a fixed external potential ν(*r*), as given Equation (13).

(13) $$f(r)={\left(\frac{\partial \rho (r)}{\partial N}\right)}_{\nu (r)}={\left(\frac{\partial \mu }{\partial \nu (r)}\right)}_{\nu (r)}$$

Roy et al. (1999) defined the electrophilic and nucleophilic Fukui functions for a site *k* in a molecule by using left and right derivatives with respect to the number of electrons as expressed *via* Equations (14–16).

(14) $$f_{k}^{+}(r)=\rho_{k}(N+1)-\rho_{k}(N)\text{ For nucleophilic attack}$$

(15) $$f_{k}^{-}(r)=\rho_{k}(N)-\rho_{k}(N-1)\text{ For electrophilic attack}$$

(16) $$f_{k}^{0}(r)=\frac{\rho_{k}(N+1)-\rho_{k}(N-1)}{2}\text{ For radical attack}$$

where ρ_*k*_(*N*), ρ_*k*_(*N–*1), and ρ_*k*_(*N*+1) are the gross electronic populations of the site *k* in neutral, cationic and anionic system, respectively.

As it is known, the concept of generalized philicity have been introduced by Chattaraj et al., they defined a local quantity called philicity associated with a site *k* in a molecule with the assistance of corresponding condensed-to-atom variants of Fukui function, $f_{k}^{\alpha }$ as in Equation (17) (Parthasarathi et al., 2004).

(17) $$\omega_{k}^{\alpha +}=\omega f_{k}^{a}$$

where α = +, − and 0 corresponds to local philic quantities describing nucleophilic, electrophilic and radical attacks, respectively. In the light of Equation (17), the highest $\omega_{k}^{\alpha +}$ corresponds to the most electrophilic site in a molecule. In addition, Lee et al. (1988) proposed different local softness, which can be used to describe the reactivity of atoms in molecules, which can be defined as in Equation (18).

(18) $$\sigma_{k}^{\alpha }=\sigma f_{k}^{\alpha }$$

where α = +, − and 0 represents local softness quantities describing nucleophilic, electrophilic and radical attacks respectively.

Recently, Morell et al. (2006) put forward a dual descriptor, Δ*f* (*k*), which is defined as the difference between the nucleophilic and electrophilic Fukui functions:

(19) $$\Delta f(k)=f_{k}^{+}-f_{k}^{-}$$

Likewise, the associated dual local softness have also been defined as expressed in Equation (20).

(20) $$\Delta \sigma_{k}=\sigma_{k}^{+}-\sigma_{k}^{-}=\sigma \Delta f_{k}$$

It is also defined as the condensed version of Δ*f*_*k*_ multiplied by the global softness σ. The multiphilic descriptor, Δω_*k*_, is defined as the difference between the nucleophilic and electrophilic condensed philicity functions. This parameter may be used as an index of selectivity toward nucleophilic attack, which can as well characterize an electrophilic attack and is given by Equation (21) (Padmanabhan et al., 2006).

(21) $$\Delta \omega_{k}=\omega \left[{\Delta f}_{k}\right]=\omega^{+}-\omega^{-}$$

If Δω_*k*_ (or Δ*f* (*k*)) > 0, the site *k* is favored for a nucleophilic attack, whereas if Δω_*k*_ (or Δ*f* (*k*)) < 0, the site *k* may be favored for an electrophilic attack.

### Molecular dynamic simulation

Adsorption characteristics of studied thiophene derivatives on iron metal surface are investigated by molecular dynamic simulation employing the Forcite module in Materials Studio 8.0 software. As model iron surface, Fe(110) surface was considered because it possesses a density packed surface and is the most stable among three common iron substrates (Guo et al., 2014). The Fe(110) system was simulated through a repeated supercell containing a 5-layer slab of Fe with 80 atoms per layer, in a 8 × 10 two-dimensional periodicity. A vacuum region 40 Å thick was included between repeated surface slabs. To simulate the metal-inhibitor systems, COMPASS force field (Sun et al., 1998) was used. The simulations of three thiophene derivatives labeled as A, B, and C on iron surface were carried out to determine the optimal adsorption sites for these molecules. All simulations made in the study were performed in an NVT canonical ensemble at 298 K with a time step of 1.0 fs and a total simulation time of 1,000 ps. The operational temperature was monitored *via* the Andersen thermostat. In the calculations, vacuum media was preferred and five layers of iron atoms were used.

To calculate the adsorption energies (*E*_ads_) on Fe(110) surface of modeled of A, B, and C molecules, we used the Equation (22). Herein, it is important to note that binding energy (*E*_binding_) is defined by the negative value of adsorption energy as given in Equation (23).

(22) $$E_{ads}=E_{\text{complex}}-\left(E_{Fe}+E_{inh}\right)$$

(23) $$E_{\text{binding}}=-E_{ads}$$

where *E*_complex_ is the total energy of an inhibitor molecule and the metal surface system. *E*_Fe_ is described as the energy of iron surface without adsorption of any inhibitor molecule and *E*_inh_ represents the energy of free inhibitor molecules.

## Results and discussion

### Global reactivity

**Figure 2:**
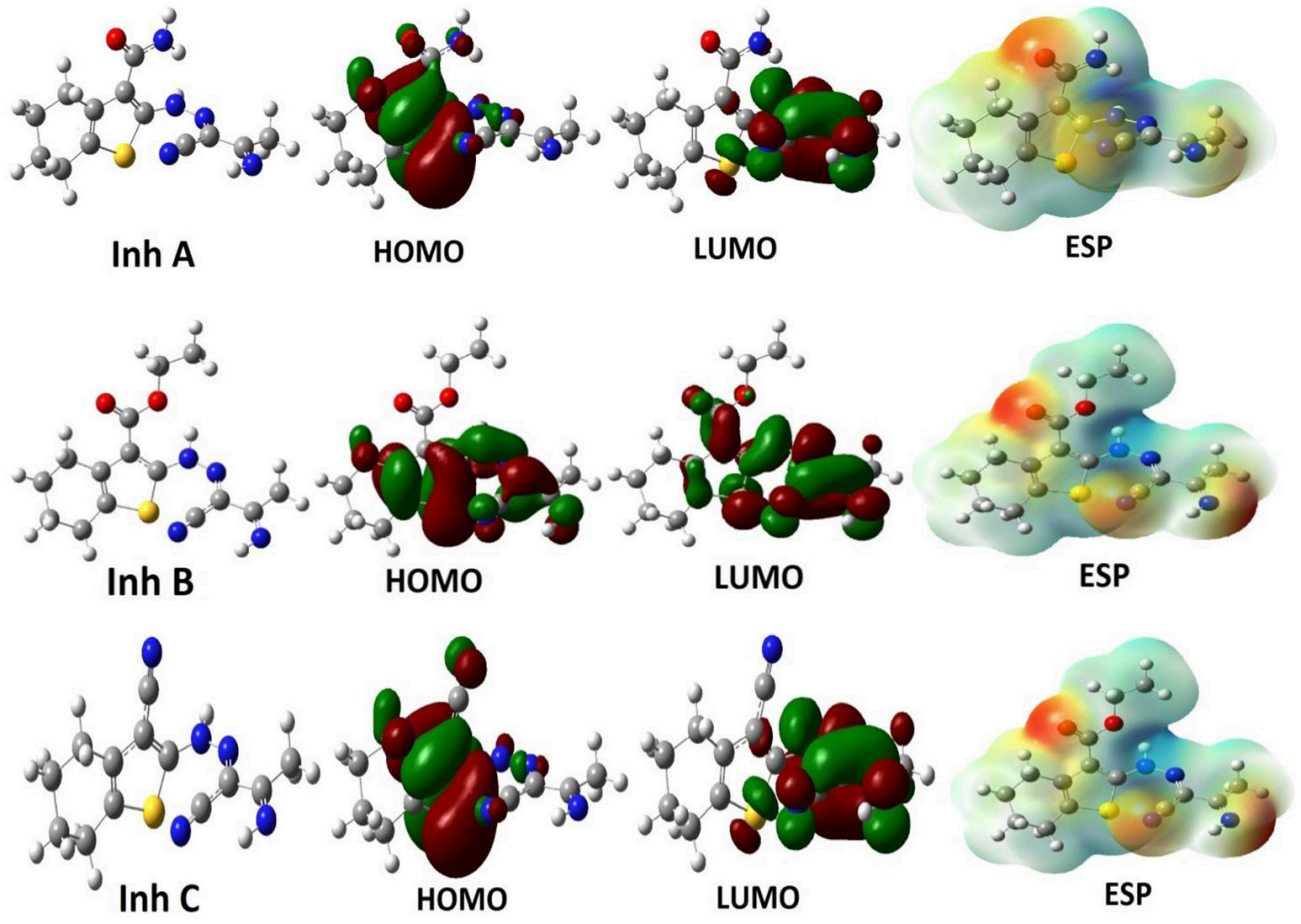
The optimized structures, HOMOs, LUMOs and electrostatic potential structures of studied inhibitor molecules at the DFT/B3LYP/6-31++G level of theory.

As mentioned in the computation section, the investigated inhibitors were optimized by performing two different methods and using three different basis sets. The optimized electronic structures correspond to energy minima with no imaginary frequencies. According to frontier molecular orbital theory and in consistent with Fukui's theory, a high *E*_HOMO_ value means the ability of a molecule to donate electrons to an assigned acceptor (metal surface in our case) with empty molecular orbital that facilitated the adsorption process and therefore indicated good inhibition performance (Obot et al., 2015). In contrast, *E*_LUMO_ is related to electron affinity, which corresponds to a tendency for electron acceptance. Accordingly, the gap between energy levels of the molecules (Δ*E* = *E*_LUMO_-*E*_HOMO_) is a significant descriptor that need be calculated. It demonstrates inveterate electron donating ability and measures the interaction between inhibitor molecules and substrate surface. The optimized molecular structures, HOMO, LUMO, as well as the molecular electrostatic potential of the investigated molecules are showed in Figure 2.

Many investigations (Khalil, 2003; Gece, 2013; Frau and Glossman-Mitnik, 2017) reported that the inhibition efficiency of inhibitors has been found to correlate with some other quantum chemical parameters like η, σ, χ, and dipole moment (DM). Besides, ω, ε, Δ*N*, Δ*E*_b-d_, Δψ, which have been calculated in terms of η and χ, are also highly useful descriptors in corrosion inhibition studies of organic molecules. Our computed quantum chemical parameters for three inhibitor molecules in gas phase and water phase are given in Tables 1, 2.

**Table 1:** Calculated quantum chemical parameters for three thiophene derivatives in gas phase (all in eV).

	***E*_HOMO_**	***E*_LUMO_**	***I***	***A***	**Δ*E***	**η**	**σ**	**χ**	**ω**	**ε**	**Energy**
**HF/SDD LEVEL**											
InhA	−8.3683	1.1344	8.3683	−1.1344	9.5028	4.7514	0.2104	3.6169	1.3766	0.7263	−33879.3080
InhB	−9.2421	1.5853	9.2421	−1.5853	10.8275	5.4137	0.1847	3.8284	1.3536	0.7387	−36540.5835
InhC	−9.7224	1.4680	9.7224	−1.4680	11.1905	5.5952	0.1787	4.1271	1.5221	0.6569	−31810.3464
**HF/6–31G LEVEL**											
InhA	−8.2290	1.3028	8.2290	−1.3028	9.5319	4.7659	0.2098	3.4630	1.2581	0.7948	−33876.6064
InhB	−8.7531	1.4999	8.7531	−1.4999	10.2530	5.1265	0.1950	3.6266	1.2827	0.7795	−36519.9142
InhC	−9.6086	1.6552	9.6086	−1.6552	11.2639	5.6319	0.1775	3.9767	1.4039	0.7122	−31808.2231
**HF/6–31**++**G LEVEL**											
InhA	−8.3387	0.7785	8.3387	−0.7785	9.1172	4.5586	0.2193	3.7801	1.5672	0.6380	−33876.6772
InhB	−9.1890	0.9053	9.1890	−0.9053	10.0944	5.0472	0.1981	4.1418	1.6994	0.5884	−36538.1638
InhC	−9.6900	0.8898	9.6900	−0.8898	10.5798	5.2899	0.1890	4.4001	1.8299	0.5464	−31808.7945
**B3LYP/SDD Level**											
InhA	5.9713	−2.6463	5.9721	2.6463	3.3258	1.6629	0.6013	4.3092	5.5834	0.1791	−34045.6874
InhB	−6.1392	−2.6893	6.1392	2.6893	3.4498	1.7249	0.5797	4.4142	5.6482	0.1770	−36723.6594
InhC	−7.0801	−2.4626	7.0801	2.4626	4.6175	2.3087	0.4331	4.7714	4.9304	0.2028	−31965.1740
**B3LYP/6–31G LEVEL**											
InhA	−5.8311	−2.4672	5.8311	2.4672	3.3639	1.6819	0.5945	4.1492	5.1178	0.1953	−34042.7085
Inh B	−5.9743	−2.5415	5.9743	2.5415	3.4327	1.7163	0.5826	4.2579	5.2814	0.1893	−36720.2907
InhC	−6.9457	−2.2580	6.9457	2.2580	4.6877	2.3438	0.4266	4.6018	4.5176	0.2213	−31962.8504
**B3LYP/6–31**++**G LEVEL**											
InhA	−6.0978	−2.7916	6.0978	2.7916	3.3062	1.6531	0.6049	4.4447	5.9753	0.1673	−34043.6827
InhB	−6.2657	−2.8027	6.2657	2.8027	3.4629	1.7314	0.5775	4.5342	5.9370	0.1684	−36721.3309
InhC	−7.2287	−2.5796	7.2287	2.5796	4.6491	2.3245	0.4301	4.9042	5.1733	0.1933	−31963.7351

**Table 2:** Calculated quantum chemical parameters for three thiophene derivatives in aqueous phase (all in eV).

	***E*_HOMO_**	***E*_LUMO_**	***I***	***A***	**Δ*E***	**η**	**σ**	**χ**	**ω**	**ε**	**Energy**
**HF/SDD LEVEL**											
InhA	−8.3123	1.0160	8.3123	−1.0160	9.3284	4.664	0.2144	3.6481	1.4267	0.7009	−33879.9951
InhB	−8.2973	1.1439	8.2973	−1.1439	9.4413	4.720	0.2118	3.5766	1.3549	0.7380	−36541.4064
InhC	−8.3999	1.1354	8.3999	−1.1354	9.5354	4.767	0.2097	3.6322	1.3835	0.7227	−31811.0425
**HF/6–31G LEVEL**											
InhA	−8.3123	1.0160	8.3123	−1.0160	9.3284	4.66420	0.2144	3.6481	1.4267	0.7009	−33879.9951
InhB	−8.1917	1.2996	8.1917	−1.2996	9.4914	4.74570	0.2107	3.4460	1.2511	0.7992	−36538.2199
InhC	−8.2886	1.2936	8.2886	−1.2936	9.5822	4.79115	0.2087	3.4975	1.2765	0.7833	−31808.9180
**HF/6–31**++**G LEVEL**											
InhA	−8.2766	0.9105	8.2766	−0.9105	9.1871	4.59359	0.2176	3.6830	1.4765	0.6772	−33877.9455
InhB	−8.2606	1.0432	8.2606	−1.0432	9.3039	4.65196	0.2149	3.6086	1.3996	0.7144	−36538.9128
InhC	−8.3648	1.0324	8.3648	−1.0324	9.3972	4.69863	0.2128	3.6662	1.4303	0.6991	−31809.5068
**B3LYP/SDD LEVEL**											
InhA	−5.9503	−2.8131	5.9503	2.8131	3.1372	1.56861	0.6375	4.3817	6.1199	0.1634	−34046.2850
InhB	−5.9658	−2.7715	5.9658	2.7715	3.1943	1.59718	0.6261	4.3686	5.9747	0.1673	−36724.3442
InhC	−6.0850	−2.8104	6.0850	2.8104	3.2746	1.63732	0.6107	4.4477	6.0410	0.1655	−31965.7859
**B3LYP/6–31G LEVEL**											
InhA	−5.8129	−2.6221	5.8129	2.6221	3.1908	1.59542	0.6268	4.2175	5.5745	0.1793	−34043.2523
InhB	−5.8385	−2.5723	5.8385	2.5723	3.2662	1.63310	0.6123	4.2054	5.4147	0.1846	−36720.9188
InhC	−5.9522	−2.6066	5.9522	2.6066	3.3456	1.67283	0.5977	4.2794	5.4738	0.1826	−31963.4123
**B3LYP/6–31**++**G level**											
InhA	−6.0336	−2.9241	6.0336	2.9241	3.1094	1.55473	0.6432	4.4788	6.4514	0.1550	−34044.2976
InhB	−6.0379	−2.8656	6.0379	2.8656	3.1723	1.58616	0.6304	4.4518	6.2473	0.1600	−36721.9827
InhC	−6.1514	−2.8972	6.1514	2.8972	3.2542	1.62712	0.6145	4.5243	6.2901	0.1589	−31964.3272

As given in Table 1, it is notable that inhibitor A has the highest *E*_HOMO_ among all the studied inhibitors. This reflects that the electron-donating ability of Inhibitor A is strong. As is known, corrosion inhibitors with low Δ*E* values provide better inhibition performances. This is because that the excitation energy to remove an electron from the last occupied orbital will be low. It was also reported that a molecule with a low energy gap could be more polarizable, which is usually associated with a high chemical reactivity and low kinetic stability, termed as soft molecule (Madkour and Elroby, 2015). Jafari et al. (2013) also pointed that adsorption of inhibitor molecule onto a metallic surface occurs at the site of the molecule which has the greatest softness and lowest hardness. Our results in Tables 1, 2 show that all the elected levels inhibitor A has the lowest Δ*E* energy in both gas and aqueous phases, and hence the molecule could have a better inhibitive performance on the iron surface as corrosion inhibitor. Based on the above discussion, we can write the corrosion inhibition efficiency (in both gas and aqueous phases) order as: A > B > C. These results are in good agreement with the available experimental results (Fouda et al., 2014). However, from the results obtained for *E*_LUMO_ in gas and aqueous phases the trend is irregular, which does not correlate well in the experimentally determination inhibition efficiency. So we claimed that LUMO energies of molecules may fail in terms of the explanation of their inhibition efficiencies.

Generally, chemical hardness (η) is the resistance against electron cloud polarization or deformation of chemical species. Thus, the η value of a molecule and its inhibition efficiency are inversely proportional to each other because a hard molecule is renitent to give electrons (Kaya et al., 2016). Lukovits et al. (2001) reported that η, σ, and Δ*E* are quantum chemical descriptors closely correlated with each other. As it is mentioned in computation part and according to Koopmans's theorem, both softness and hardness are obtained on the basis of HOMO and LUMO orbital energies. Hard molecules with high Δ*E* cannot act as good corrosion inhibitor. Nevertheless, soft molecules which have low Δ*E* could be excellent corrosion inhibitors since they can easily donate electrons to metals. Based on the results reported in Tables 1, 2, it is clear that the sequence of inhibitive efficiency for studied molecules based on their hardness, and softness values can be written as: A > B > C.

In the light of the simple charge transfer theory for donation and back-donation of charges proposed by Gomez et al. (2006), the electronic back-donation process can probably affect the interaction between the inhibitors and substrate surface. As given in Equation (11), when the electron transfer and back-donation processes occur simultaneously, the energy change is directly proportional to the hardness of the inhibitor molecule. The Δ*E*_b−d_ indicates that since η > 0, then Δ*E*_b−d_ < 0, and the charge transfer from a molecule, followed by a back-donation to the molecule, is energetically favored (Bedair, 2016). Based on this principle, it is available to compare the stabilization among the inhibitor molecules, because there will be an interaction with the same metal, it is obvious that Δ*E*_b−d_ will decrease with the hardness increases. According to our results given in Table 3, as expected and in agreement with the experimental results (Fouda et al., 2014), the calculated Δ*E*_b−d_ exhibit the tendency: A > B > C.

**Table 3:** Calculated Δ*E*_*b*−*d*_ (back-donation), Δ*N* (the fraction of electrons transferred), Δψ (the initial molecule-metal interaction energy), and dipole moment (DM) values for studied thiophene derivatives in gas phase and aqueous phase.

	**Gas phase**					**Aqueous phase**			
	**Δ*N***	**Δψ**	**Δ*E*_b−d_**	**DM**		**Δ*N***	**Δψ**	**Δ*E*_b−d_**	**DM**
**HF/SDD LEVEL**									
InhA	0.3560	−0.6022	−1.1879	9.4486	InhA	0.3626	−0.6206	−1.1661	11.4028
InhB	0.2929	−0.4645	−1.3534	5.6345	InhB	0.3593	−0.6022	−1.1802	10.0481
InhC	0.2567	−0.3688	−1.3988	6.5411	InhC	0.3532	−0.5947	−1.1919	7.0885
**HF/6-31G LEVEL**									
InhA	0.3711	−0.6562	−1.1915	9.1042	InhA	0.3744	−0.6654	−1.1661	10.9218
InhB	0.3291	−0.5549	−1.2816	5.5724	InhB	0.3590	−0.6548	−1.1864	9.9318
InhC	0.2684	−0.4057	−1.4080	6.3800	InhC	0.3655	−0.6401	−1.1978	6.9518
**HF/6-31**++**G LEVEL**									
InhA	0.3532	−0.5686	−1.1397	5.2333	InhA	0.3645	−0.6181	−1.1484	11.8212
InhB	0.2831	−0.4046	−1.2618	5.8830	InhB	0.3610	−0.6014	−1.1630	10.1740
InhC	0.2457	−0.3195	−1.3225	6.5714	InhC	0.3548	−0.5914	−1.1747	7.3299
**B3LYP/SDD LEVEL**									
InhA	0.8091	−1.0885	−0.4157	10.2193	InhA	0.8346	−1.0926	−0.3922	9.9536
InhB	0.7495	−0.9690	−0.4312	5.6910	InhB	0.8237	−1.0838	−0.3993	10.2031
InhC	0.4826	−0.5378	−0.5772	6.8723	InhC	0.7794	−0.9946	−0.4093	8.0342
**B3LYP/6-31G LEVEL**									
InhA	0.8475	−1.2080	−0.4205	9.5972	InhA	0.872	−1.2132	−0.3989	9.6734
InhB	0.7988	−1.0952	−0.4291	5.2711	InhB	0.8556	−1.1955	−0.4083	9.8580
InhC	0.5116	−0.6134	−0.5860	6.5282	InhC	0.8132	−1.1061	−0.4182	7.5795
**B3LYP/6-31**++**G LEVEL**									
InhA	0.7729	−0.9874	−0.4133	10.149	InhA	0.8108	−1.0234	−0.3887	10.2794
InhB	0.7120	−0.8778	−0.4329	5.6794	InhB	0.8033	−1.0210	−0.3965	10.3650
InhC	0.4508	−0.4724	−0.5811	6.9109	InhC	0.7608	−0.9417	−0.4068	8.3739

Electrophilicity index (ω) represents the propensity of a molecule to receive electrons. Conversely, nucleophilicity (ε) indicates the tendency to donate or share electrons with others, it is defined as the inverse of electrophilicity (1/ω). It is generally assumed that a molecule that has a large electrophilicity value is ineffective against corrosion while a molecule that has a large nucleophilicity value is expected to an excellent candidate as corrosion inhibitor. Based on Tables 1, 2, it is evident that the inhibiors have low electrophilicity index values and are good nucleophiles. But there exists discrepancy in trend between the HF and B3LYP functionals for electrophilicity, which is due to the quadratic dependence on the electronegativity. In view of this, their molecular reactivity cannot be predicted accurately, it is necessary to take into account another additional criterion to determine their inhibitive capacity.

In this work, the number of electrons transferred (Δ*N*) of the between metal substrate and inhibitor molecules was calculated using Equation (10). The results are also gathered in Table 3. Based on the Sanderson's electronegativity equalization principle (Sanderson, 1983), the charge transfer process between metal and inhibitor will continue until their electronegativity values are equal with each other. In fact, Δ*N* can be regarded as a derived descriptor from the electronegativity/hardness equalization principle. It was pointed out that the positive value of electrons transferred (Δ*N*) indicates that the molecules act as electron donors. Based on the tabulated results (Table 3), we can see that molecule **A** has the largest number of the fraction of transferred electrons (Δ*N*) in both gas and aqueous phases, turn is, A > B > C, regardless of phase and the elected levels. These results agree well with the experimental results. Another important parameter is the initial molecule-metal interaction energy (Δψ), which has been introduced in Kokalj's work (Kovacevic and Kokalj, 2011). In our work, we have calculated (Δψ) for all the researched inhibitors and the results are given in Table 3. The results indicate that the trend of Δψ is also A > B > C.

Recently, some authors used the dipole moment as an indicator of corrosion inhibition efficiencies of molecules (Gece, 2008; Zarrouk et al., 2014). Several authors showed that corrosion inhibition efficiency increases with the increase of dipole moment (Stoyanova et al., 2002; Sahin et al., 2008). Considering the idea that increasing value of dipole moment facilitates the electron transport process. Yet others proposed the opposite correlation, that is, a low value of dipole moment favors the accumulation of inhibitor molecules on the metal surface and ultimately increasing the inhibition performances (Khalil, 2003; Lebrini et al., 2005). As shown in Table 3, the calculated the dipole moments for studied compounds are irregular. There is no any remarkable relationship between dipole moment and inhibition efficiency. Thus it cannot be used a priori to judge the inhibition effectiveness.

Overall, based on the global descriptors considered for each molecule shown in Tables 1–3, The order of chemical activities from high to low is: –C(=O)–NH_2_ > –C(=O)–OC_2_H_5_ > –C≡N substituents. Correspondingly, the inhibitive effectiveness order for the thiophene molecules is: InhA > InhB > InhC. This indicates that our calculated theoretical results are in agreement with experimental orders.

### Local reactivity

In order to have an understanding on the local reactivity of the thiophene derivatives, the Fukui indices for every atom in the inhibitors have been calculated at the B3LYP/6-31++G level. It is well known that an analysis of the Fukui indices and the local descriptors provides a more comprehensive information of the reactivity of the molecules under probe. To complete the picture, the local softness, local electrophilicity, and the dual descriptors have been also calculated for each atoms in the studied molecules.

**Figure 3:**
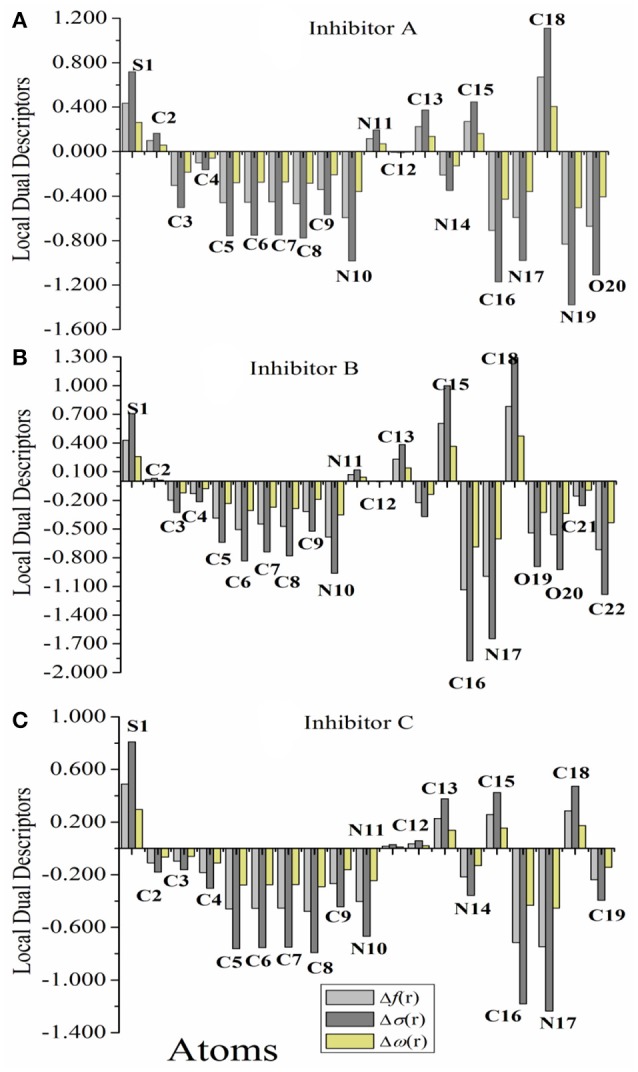
**(A–C)** Graphical representation of the local dual descriptors, Δ*f*, Δσ, and Δω, based on Fukui Functions of the studied inhibitors A, B, and C (the atom-numbering is in correspond with Figure 1).

Figure 3 represents graphically the local dual descriptors, Δ*f*_*k*_, Δσ_*k*_, and Δω_*k*_ for the three compounds. It should be noted that the numbering of atoms, which is given in Figure 1 are employed in this analysis. Generally, the condensed Fukui functions can make us to distinguish each part of the inhibitor molecule in the light of its distinct chemical behavior with different substituent functional groups. Therefore, the site for nucleophilic attack will be the place where the value of *f*^−^ is a maximum. Conversely, the site for electrophilic attack is controlled by the value of *f*^+^.

The results reported in Tables S1–S3 reveal that, for nucleophilic attack, the highest $f_{k}^{-}$ values of InhA are N19, N17, O20, and C16 atoms. For InhB, the most nucleophilic sites are N17, C16, and C22 atoms. For InhC, the most reactive sites are C16 and N17. This indicates the propensity to donate electrons to vacant molecular orbital on the iron surface to form coordinate bond. This agrees with the results of the computed HOMO density. For electrophilic attack, the highest $f_{k}^{-}$ values of the three studied inhibitors are S1, C18, C15, and C13, indicating that the sites most capable for an electrophilic attack that is through which the molecule accepts electrons to form feedback bonds with Fe(110) surface. This also conforms to the computed LUMO orbital density. These results are also supported by the values of the local dual indices (Δ*f*, Δσ, and Δω), which indicate that these inhibitors have many active sites and most of these centers have values of the three descriptors of lower than 0, except some atoms, which found to be have values > 0 (see Figure 3), indicating an electrophilic centers. A close inspection would reveal that all the molecules had the back-donation process at their carbon atoms in agreement with the frontier orbital results obtained. According to these results, one can conclude that Inh A molecule will have many active centers to interact with iron substrate. These are most likely those areas that containing N and O atoms, which are the most possible sites for bonding to iron surface through donating electrons to the Fe 3*d* orbitals (Khaled, 2010). Also, it can be suggested that the binding between the surface of the metal with the InhA is stronger than that in the case of B and C, respectively. Finally, the above local descriptors reveal that the theoretical order for the variation of inhibition efficiencies of the investigative inhibitors agrees with the available experimental data and it is as follows: A > B > C.

### PCA and AHCA analysis

In this work, all calculated variables have been auto scaled to compare them at the same level. Thereafter, principal component analysis (PCA) was adopted to reduce the number of variables and select the most relevant ones, which are responsible for the reactivity of researched thiophene derivatives. After performing many tests, a good separation was obtained between more active and less active thiophene compounds using 11 variables: *E*_HOMO_, *I*, Δ*E*, χ, μ, σ, ω, Δ*N*, Δψ, and Δ*E*_b−d_. As indicated from PCA results, the first two principal components (F1 and F2) describe all of the overall variance as follows: F1 = 84.58% and F2 = 15.42%. The score plot of the variances is a reliable representation of the spatial distribution of the points for the data set studied after explaining almost all of the variances by the first two principal components.

**Figure 4:**
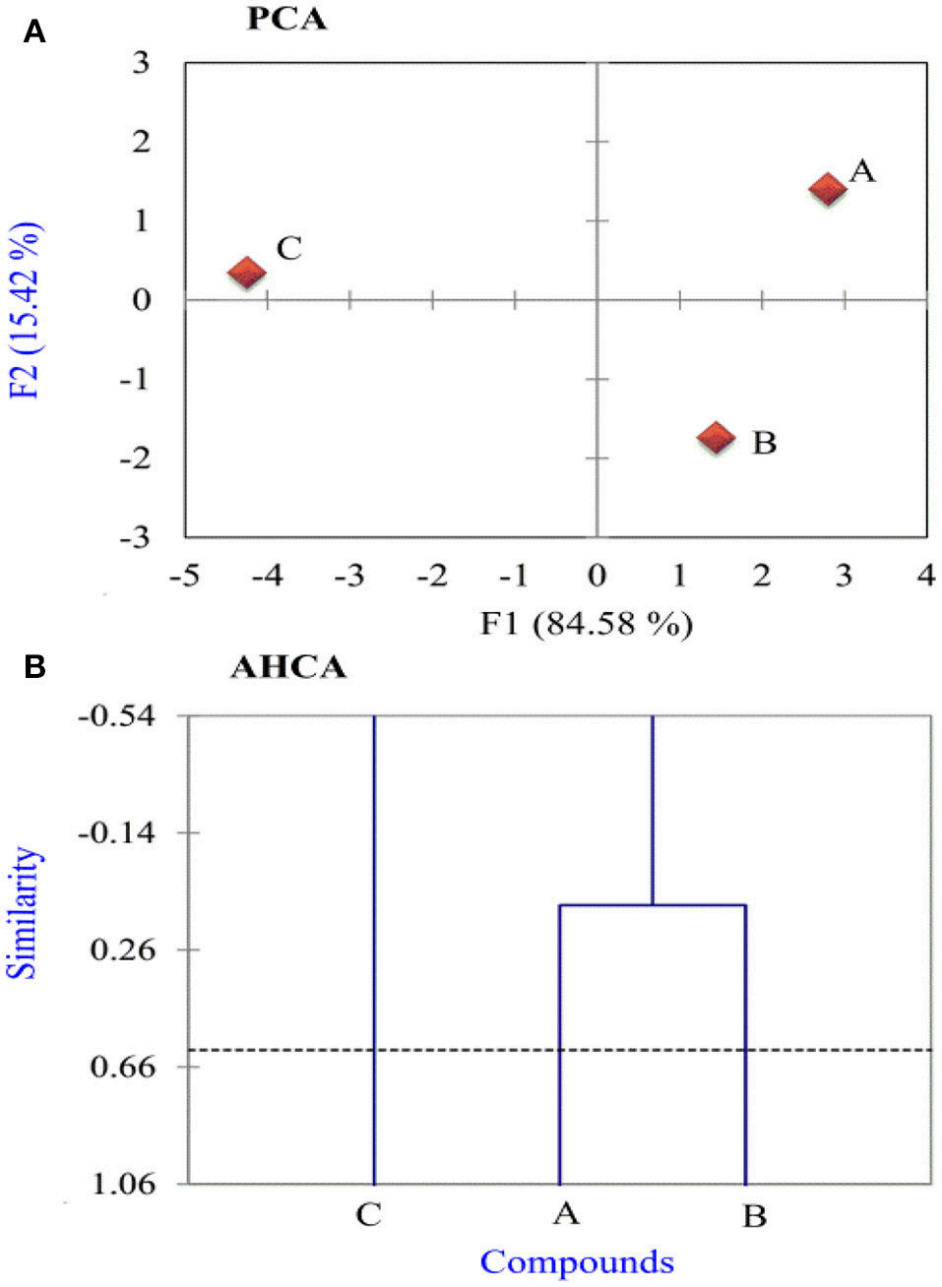
**(A)** Score and **(B)** dendrogram plots obtained for three studied inhibitors.

In Figure 4A, the most informative score plot for the inhibitors is presented (F1 vs. F2). It is evident from the figure that PCA is responsible for the separation between more active InhA and InhB and less active Inh C where F1 > 0 for the more active compounds, and F1 < 0 for the less active one. These results are in a well agreement with the experimental results, the calculated global and local descriptors. Figure 4B shows AHCA analysis for the inhibitors under probe. The horizontal lines represent the inhibitors and the vertical lines the similarity values between pairs of inhibitors, an inhibitor and a group of inhibitors and among groups of inhibitors. It is noticed that AHCA results are very similar to those obtained with the PCA analysis, i.e., the studied inhibitors were grouped into two categories: More active inhibitors A and B and less active one C.

### Molecular dynamic simulations

**Figure 5:**
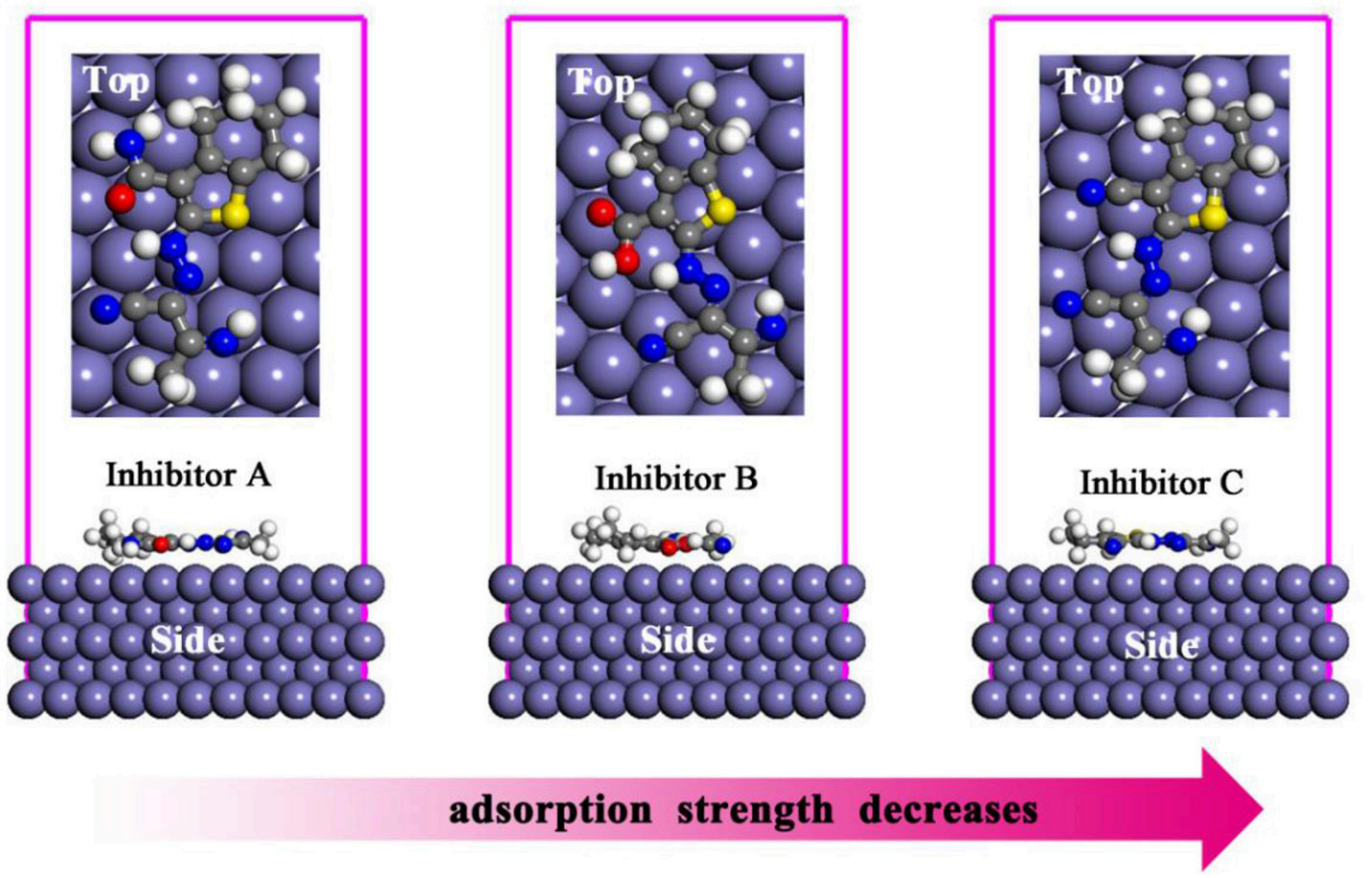
Side and top views of the most stable adsorption models of three inhibitors on Fe(110) surfaces.

Molecular dynamic simulation approach is very important in terms of the explanation of the nature of the interactions between corrosion inhibitors and metallic surface. The optimized equilibrium adsorption configurations for A, B, and C molecules on Fe(110) surface are given in Figure 5. Adsorption energy is known as the energy released when inhibitor molecule was adsorbed on metal surface. As given in previous section, the binding energy is the negative value of the adsorption energy. Higher negative value of adsorption energy and higher positive values of binding energy represent the more stable and more strong interaction between metal surface and inhibitor molecule. In Table 4, calculated adsorption and binding energies as well as experimentally determined corrosion inhibition efficiencies for studied thiophene derivatives are presented. It is apparent that the binding energies of three derivatives on the Fe(110) substrate decrease in the order A > B > C, which is in consonance with the experimental inhibition efficiency orders (Fouda et al., 2014).

**Table 4:** Experimental inhibition efficiency, *IE*(%), as well as the outputs and descriptors obtained from MD simulation for the adsorption of A, B, and C on Fe(110) surface.

**Systems**	***E*_ads_ (kJ·mol^−1^)**	***E*_binding_ (kJ·mol^−1^)**	***IE*%**
A+Fe(110)	−658.0	658.0	91.7
B+Fe(110)	−649.7	649.7	90.6
C+Fe(110)	−613.1	613.1	85.7

### Comparison between experimental and theoretical results

**Figure 6:**
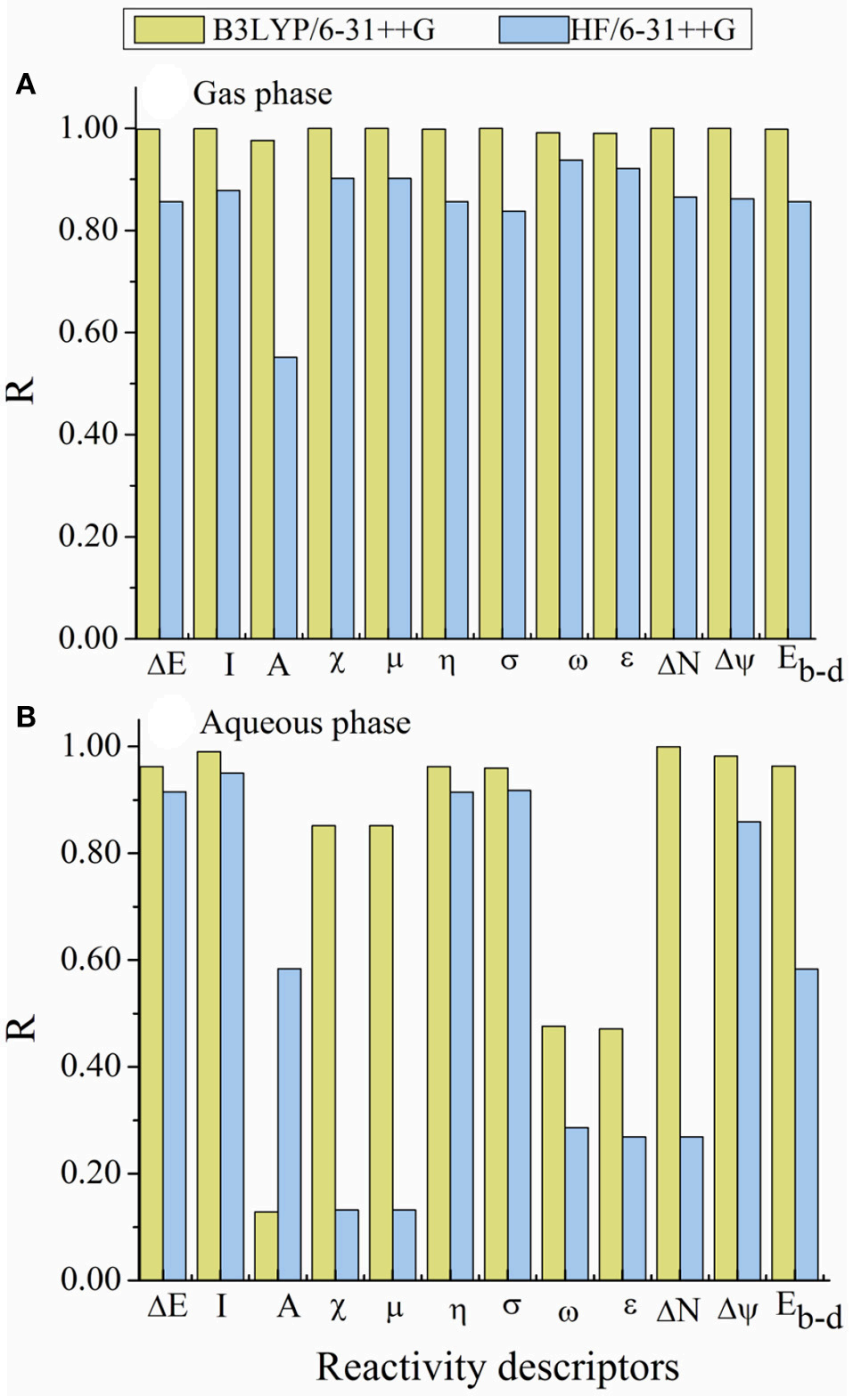
Graphical representation of the linear relationship coefficient (*R*) obtained between the calculated reactivity descriptors and the experimental inhibition efficiency (*IE*%). **(A)** in gas phase and **(B)** in aqueous phase.

In this subsection, a comparison between experimental and theoretical results is presented. Our study shows that there is an excellent correlation between our theoretical results (global quantum descriptors and MDs results) with the experimental inhibition efficiency (*IE*%). Figures 6A,B shows graphical representation of the relationship between the linear correlation *R* obtained for the relationship between theoretical reactivity parameters calculated (in both gas and aqueous phases) using B3LYP and HF methods with 6-31++G basis sets for the studied inhibitors and their experimental *IE*%. The results of linear relationship coefficients are summarized in Table S4. However, the results obtained *via* B3LYP method are well correlated with the experimental results than those obtained by HF method.

**Figure 7:**
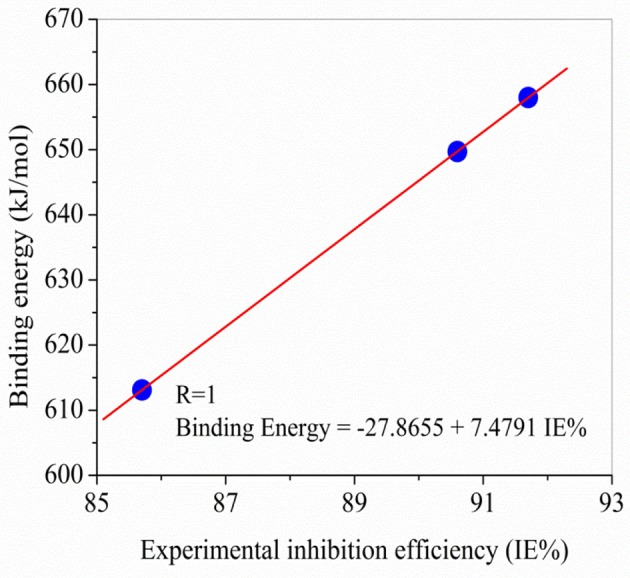
Comparisons made between experimental anticorrosion efficiencies and theoretical binding energy obtained in the study.

As can be seen in Figure 6, there are a very excellent linear correlation between the experimental inhibition efficiency and the theoretical descriptors in gas phase for both methods in gas phase. It is apparent from the column graphs plotted that B3LYP method provided more accurate results compared to HF method. In addition, it can be said that calculation levels including 6-31++G basis set is more successful compared to other calculation levels in terms of the obtaining good agreement with experimental results. Finally, it can also be seen from Figure 7 that there exists closely correlation between experimental anticorrosion efficiencies and calculated binding energies with a high correlation coefficient of 1.00.

## Conclusions

In this work, Hartree Fock as well as DFT calculations, molecular dynamic simulation, PCA, and AHCA were used to analyze the anticorrosive performances of some thiophene derivatives against the iron metal. Global and local reactivity descriptors were calculated in both gas and aqueous phase for three studied inhibitors. Within the framework of the theoretical results obtained in this study, the following conclusions can be written.

1. DFT, molecular dynamic simulation, PCA, and AHCA results showed that corrosion inhibition efficiency ranking of studied molecules is given as: InhA > InhB > InhC.
2. It is apparent from binding energies and adsorption energies calculated for studied thiophene derivatives, these molecules are very effective against the corrosion of iron.
3. According the PCA and AHCA results, least active inhibitor among the studied molecules is inhibitor C.
4. Both theoretical data and experimental results are compatible with inductive effect of functional groups appearing in the molecular structures of studied thiophene derivatives.
5. Theoretical results obtained in this work have far-reaching significance to the rational design of novel thiophene derivatives as corrosion inhibitor.

## Author contributions

LG, ZS, and SK performed all the calculations. WS, BT, NA, and CK were involved in conception and design of the experiments. All authors were involved the drafting, revision and approval of the manuscript.

## Conflict of interest statement

The authors declare that the research was conducted in the absence of any commercial or financial relationships that could be construed as a potential conflict of interest.
