# Supplementary material for: Anticorrosive Effects of Some Thiophene Derivatives Against the Corrosion of Iron: A Computational Study
Source: Front Chem. 2018 May 7;6:155. doi: 10.3389/fchem.2018.00155 (PMC5949371; doi:10.3389/fchem.2018.00155)
Supplement: Supplementary file 1 [file Table_1.DOC]

Supplementary Material

Anticorrosive Effects of Some Thiophene Derivatives Against the Corrosion of Iron: A Computational Study

***Lei Guo*******,*** ***Zaki S. Safi, Savaş Kaya, Wei Shi*, Burak Tüzün******, Nail Altunay*** ***and Cemal Kaya***

****Correspondence:*** *cqglei@163.com; truweishi@163.com*

| **TABLE S1.** Fukui indices for Inhibitor A. | | | | | | | | | |
| --- | --- | --- | --- | --- | --- | --- | --- | --- | --- |
|  | *f^+^* | *f^-^* | *σ^+^* | *σ^−^* | ω^+^ | ω^-^ | Δ*f* | Δσ | Δω |
| **S1** | 0.2567 | -0.1784 | 0.4244 | -0.2948 | 0.1553 | -0.1079 | 0.4351 | 0.7192 | 0.2632 |
| **C2** | 0.1029 | 0.0029 | 0.1701 | 0.0048 | 0.0622 | 0.0018 | 0.0999 | 0.1652 | 0.0605 |
| **C3** | −0.0593 | 0.2451 | -0.0980 | 0.4052 | -0.0359 | 0.1483 | -0.3044 | -0.5032 | -0.1841 |
| **C4** | −0.0369 | 0.0621 | -0.0610 | 0.1027 | -0.0223 | 0.0376 | -0.0990 | -0.1637 | -0.0599 |
| **C5** | −0.2347 | 0.2236 | -0.3880 | 0.3696 | -0.1420 | 0.1352 | -0.4583 | -0.7576 | -0.2772 |
| **C6** | -0.2299 | 0.2253 | -0.3800 | 0.3724 | -0.1391 | 0.1363 | -0.4552 | -0.7525 | -0.2753 |
| **C7** | -0.2276 | 0.2248 | -0.3763 | 0.3715 | -0.1377 | 0.1360 | -0.4524 | -0.7478 | -0.2737 |
| **C8** | -0.2468 | 0.2229 | -0.4080 | 0.3685 | -0.1493 | 0.1348 | -0.4697 | -0.7765 | -0.2841 |
| **C9** | -0.0379 | 0.3045 | -0.0627 | 0.5033 | -0.0229 | 0.1842 | -0.3424 | -0.5659 | -0.2071 |
| **N10** | -0.2259 | 0.3681 | -0.3734 | 0.6086 | -0.1366 | 0.2227 | -0.5940 | -0.9820 | -0.3593 |
| **N11** | 0.1485 | 0.0310 | 0.2455 | 0.0512 | 0.0898 | 0.0187 | 0.1175 | 0.1943 | 0.0711 |
| **C12** | 0.1347 | 0.1411 | 0.2226 | 0.2332 | 0.0815 | 0.0853 | -0.0064 | -0.0106 | -0.0039 |
| **C13** | 0.1175 | -0.1534 | 0.1943 | -0.2535 | 0.0711 | -0.0928 | 0.2709 | 0.4478 | 0.1639 |
| **N14** | 0.0026 | 0.2136 | 0.0043 | 0.3531 | 0.0016 | 0.1292 | -0.2110 | -0.3487 | -0.1276 |
| **C15** | 0.0922 | -0.1347 | 0.1524 | -0.2226 | 0.0558 | -0.0815 | 0.2269 | 0.3750 | 0.1372 |
| **C16** | -0.3562 | 0.3519 | -0.5889 | 0.5817 | -0.2155 | 0.2129 | -0.7081 | -1.1705 | -0.4283 |
| **N17** | -0.1647 | 0.4279 | -0.2723 | 0.7073 | -0.0996 | 0.2588 | -0.5926 | -0.9796 | -0.3585 |
| **C18** | 0.3477 | -0.3238 | 0.5747 | -0.5352 | 0.2103 | -0.1958 | 0.6714 | 1.1099 | 0.4062 |
| **N19** | -0.3879 | 0.4452 | -0.6412 | 0.7359 | -0.2346 | 0.2693 | -0.8330 | -1.3771 | -0.5039 |
| **O20** | -0.3044 | 0.3666 | -0.5033 | 0.6061 | -0.1842 | 0.2218 | -0.6711 | -1.1093 | -0.4059 |

| **TABLE S2**. Fukui indices for inhibitor B | | | | | | | | | |
| --- | --- | --- | --- | --- | --- | --- | --- | --- | --- |
| **Atom** | *f^+^* | *f^-^* | *σ^+^* | *σ^−^* | ω^+^ | ω^-^ | Δ*f* | Δσ | Δω |
| **S1** | 0.2565 | -0.1719 | 0.4241 | -0.2842 | 0.1552 | -0.1040 | 0.4284 | 0.7082 | 0.2592 |
| **C2** | 0.0804 | 0.0619 | 0.1328 | 0.1023 | 0.0486 | 0.0374 | 0.0185 | 0.0305 | 0.0112 |
| **C3** | -0.0473 | 0.1505 | -0.0782 | 0.2487 | -0.0286 | 0.0910 | -0.1978 | -0.3269 | -0.1196 |
| **C4** | -0.0346 | 0.0943 | -0.0572 | 0.1559 | -0.0209 | 0.0571 | -0.1289 | -0.2132 | -0.0780 |
| **C5** | -0.2124 | 0.1732 | -0.3512 | 0.2863 | -0.1285 | 0.1048 | -0.3856 | -0.6374 | -0.2333 |
| **C6** | -0.2362 | 0.2679 | -0.3905 | 0.4428 | -0.1429 | 0.1620 | -0.5041 | -0.8333 | -0.3049 |
| **C7** | -0.2280 | 0.2187 | -0.3769 | 0.3615 | -0.1379 | 0.1323 | -0.4467 | -0.7384 | -0.2702 |
| **C8** | -0.2476 | 0.2240 | -0.4092 | 0.3703 | -0.1497 | 0.1355 | -0.4716 | -0.7796 | -0.2853 |
| **C9** | -0.0310 | 0.2840 | -0.0512 | 0.4694 | -0.0187 | 0.1718 | -0.3149 | -0.5206 | -0.1905 |
| **N10** | -0.2194 | 0.3631 | -0.3627 | 0.6002 | -0.1327 | 0.2196 | -0.5825 | -0.9630 | -0.3524 |
| **N11** | 0.1246 | 0.0521 | 0.2059 | 0.0862 | 0.0753 | 0.0315 | 0.0724 | 0.1197 | 0.0438 |
| **C12** | 0.1134 | 0.1159 | 0.1875 | 0.1916 | 0.0686 | 0.0701 | -0.0025 | -0.0041 | -0.0015 |
| **C13** | 0.0950 | -0.1366 | 0.1570 | -0.2259 | 0.0575 | -0.0827 | 0.2316 | 0.3829 | 0.1401 |
| **N14** | -0.0115 | 0.2112 | -0.0191 | 0.3492 | -0.0070 | 0.1278 | -0.2228 | -0.3682 | -0.1348 |
| **C15** | 0.1194 | -0.4853 | 0.1974 | -0.8022 | 0.0722 | -0.2935 | 0.6047 | 0.9996 | 0.3658 |
| **C16** | -0.3558 | 0.7801 | -0.5881 | 1.2895 | -0.2152 | 0.4719 | -1.1358 | -1.8776 | -0.6871 |
| **N17** | -0.1833 | 0.8119 | -0.3031 | 1.3422 | -0.1109 | 0.4912 | -0.9953 | -1.6453 | -0.6021 |
| **C18** | 0.4289 | -0.3545 | 0.7090 | -0.5860 | 0.2595 | -0.2145 | 0.7834 | 1.2951 | 0.4739 |
| **O20** | -0.2160 | 0.3233 | -0.3571 | 0.5345 | -0.1307 | 0.1956 | -0.5393 | -0.8916 | -0.3262 |
| **O19** | -0.2840 | 0.2739 | -0.4694 | 0.4529 | -0.1718 | 0.1657 | -0.5579 | -0.9223 | -0.3375 |
| **C21** | -0.0647 | 0.0894 | -0.1070 | 0.1478 | -0.0391 | 0.0541 | -0.1541 | -0.2548 | -0.0932 |
| **C22** | -0.3496 | 0.3665 | -0.5780 | 0.6059 | -0.2115 | 0.2217 | -0.7162 | -1.1839 | -0.4332 |

| **TABLE S3.** Fukui indices for inhibitor C | | | | | | | | | |
| --- | --- | --- | --- | --- | --- | --- | --- | --- | --- |
| **Atom** | *f^+^* | *f^-^* | *σ^+^* | *σ^−^* | ω^+^ | ω^-^ | Δ*f* | Δσ | Δω |
| **S1** | 0.2946 | -0.1957 | 0.4870 | -0.3235 | 0.1782 | -0.1184 | 0.4903 | 0.8105 | 0.2966 |
| **C2** | 0.0892 | 0.1986 | 0.1475 | 0.3283 | 0.0540 | 0.1201 | -0.1094 | -0.1808 | -0.0661 |
| **C3** | -0.0277 | 0.0704 | -0.0457 | 0.1163 | -0.0167 | 0.0426 | -0.0980 | -0.1620 | -0.0593 |
| **C4** | -0.0351 | 0.1475 | -0.0580 | 0.2438 | -0.0212 | 0.0892 | -0.1826 | -0.3018 | -0.1105 |
| **C5** | -0.2351 | 0.2261 | -0.3886 | 0.3738 | -0.1422 | 0.1368 | -0.4612 | -0.7625 | -0.2790 |
| **C6** | -0.2306 | 0.2254 | -0.3812 | 0.3726 | -0.1395 | 0.1363 | -0.4560 | -0.7538 | -0.2758 |
| **C7** | -0.2276 | 0.2261 | -0.3762 | 0.3738 | -0.1377 | 0.1368 | -0.4537 | -0.7500 | -0.2744 |
| **C8** | -0.2495 | 0.2300 | -0.4124 | 0.3802 | -0.1509 | 0.1391 | -0.4795 | -0.7926 | -0.2900 |
| **C9** | -0.0101 | 0.2586 | -0.0168 | 0.4274 | -0.0061 | 0.1564 | -0.2687 | -0.4442 | -0.1626 |
| **N10** | -0.1878 | 0.2162 | -0.3105 | 0.3574 | -0.1136 | 0.1308 | -0.4040 | -0.6679 | -0.2444 |
| **N11** | 0.1291 | 0.1113 | 0.2134 | 0.1840 | 0.0781 | 0.0673 | 0.0178 | 0.0294 | 0.0108 |
| **C12** | 0.0665 | 0.0303 | 0.1099 | 0.0501 | 0.0402 | 0.0183 | 0.0362 | 0.0599 | 0.0219 |
| **C13** | 0.1089 | -0.1200 | 0.1800 | -0.1983 | 0.0659 | -0.0726 | 0.2289 | 0.3783 | 0.1384 |
| **N14** | -0.0422 | 0.1741 | -0.0697 | 0.2878 | -0.0255 | 0.1053 | -0.2163 | -0.3575 | -0.1308 |
| **C15** | 0.1309 | -0.1263 | 0.2164 | -0.2087 | 0.0792 | -0.0764 | 0.2572 | 0.4251 | 0.1556 |
| **C16** | -0.3565 | 0.3581 | -0.5893 | 0.5920 | -0.2157 | 0.2166 | -0.7146 | -1.1813 | -0.4323 |
| **N17** | -0.2043 | 0.5434 | -0.3377 | 0.8982 | -0.1236 | 0.3287 | -0.7476 | -1.2359 | -0.4523 |
| **C18** | 0.1363 | -0.1498 | 0.2254 | -0.2476 | 0.0825 | -0.0906 | 0.2861 | 0.4729 | 0.1731 |
| **N19** | -0.0549 | 0.1831 | -0.0907 | 0.3028 | -0.0332 | 0.1108 | -0.2380 | -0.3935 | -0.1440 |

| **TABLE S4.** Linear relationship coefficients between experimental inhibition efficiency (IE%) and the theoretical global descriptors calculated for the studied inhibitor using B3LYP and HF methods with 6-31++G basis set in both gas and aqueous phases. | | | | | |
| --- | --- | --- | --- | --- | --- |
| Reactivity index | B3LYP/6-31++G | |  | HF/6-31++G | |
|  | Gas | Aqueous |  | Gas | Aqueous |
| Δ*E* | 0.9978 | 0.9626 |  | 0.9153 | 0.8567 |
| *I* | 0.9994 | 0.9902 |  | 0.9502 | 0.8782 |
| *A* | 0.9764 | 0.1283 |  | 0.5832 | 0.5517 |
| *χ* | 0.9999 | 0.8515 |  | 0.1318 | 0.9019 |
| *μ* | 0.9999 | 0.8515 |  | 0.1318 | 0.9019 |
| *η* | 0.9978 | 0.9622 |  | 0.9150 | 0.8565 |
| *σ* | 0.9996 | 0.9594 |  | 0.9174 | 0.8374 |
| *ω* | 0.9915 | 0.4762 |  | 0.2857 | 0.9379 |
| *ε* | 0.9904 | 0.4709 |  | 0.2687 | 0.9212 |
| Δ*N* | 1.0000 | 0.9994 |  | 0.2687 | 0.8656 |
| Δ*ψ* | 0.9995 | 0.9824 |  | 0.8591 | 0.8621 |
| Δ*E*_b-d_ | 0.9978 | 0.9635 |  | 0.5829 | 0.8566 |
